# 3D Measurements of Lubricant and Surface Temperatures Within an Elastohydrodynamic Contact

**DOI:** 10.1007/s11249-017-0953-2

**Published:** 2017-11-27

**Authors:** Jia Lu, Tom Reddyhoff, Daniele Dini

**Affiliations:** Tribology Group, Department of Mechanical Engineering, Imperial College London, South Kensington Campus, Exhibition Road, London, SW7 2AZ UK

**Keywords:** Elastohydrodynamic (EHD) lubrication, Infrared microscopy, Temperature measurements, Rheology

## Abstract

We present an infrared microscopy technique, capable of measuring the temperature of both the bounding surfaces and the oil film in an elastohydrodynamic contact. This technique can, for the first time, spatially resolve the oil film temperature in three dimensions. The contact is produced by loading a steel ball against a sapphire disc, and the film is viewed using an infrared microscope focussing through the disc. Two band pass filters are used to isolate the radiation from the oil film, and Planck’s law is applied to data obtained at a known temperature as part of the calibration procedure. The proposed technique requires the emissivity of the oil film to be measured, which is acquired in situ and is shown to vary strongly as a function of thickness and temperature. The technique is validated under pure rolling conditions, when the temperature of the oil film is equal to the controlled lubricant reservoir temperature, and also compared to an equation commonly used to predict average film temperatures, confirming the value of the unknown constant. The technique is then used to gain insights into the thermal/rheological behaviour within a contact. This is important since the temperature of elastohydrodynamic contacts is critical in determining friction and hence the efficiency of machine components and this technique enables much needed validation and provides input data for CFD and numerical simulations.

## Introduction

Frictional heat generation in lubricated contacts has been a major area of research during the last 50 years as it affects the performance of materials in industrial applications. The resulting temperature rise can lead to materials melting [1], cracking [2], oxidising [3, 4] or deforming [5], especially under dry conditions. Under lubricated conditions, temperature rises caused by friction can affect fluid properties such as film thickness, traction, shear stress, additive reaction and can even cause degradation [6] or film failure [7]. Temperature as the major detection index of heat generation, therefore, plays a significant role in tribology research [8, 9]. In addition to the problems described above, temperature rises caused by friction can provide useful information to understand and predict the behaviour of contacting surfaces and lubricants, since temperature variations are closely related with the mechanisms of friction and heat dissipation. For example, temperatures measured in shear heating experiments can be used to validate contact temperature values [10, 11] or elucidate friction mechanisms [12].

An area where detailed interfacial temperature measurements are particularly needed is elastohydrodynamic (EHD) lubrication, which occurs in the contacts between, e.g. rolling element bearings, gears and cams. Under normal operating conditions, the energy efficiency of these components is largely determined by shearing of the lubricant film that separates the sliding surfaces. However, this is difficult to predict and hence optimise, since the non-conformal nature of component surfaces results in contact widths of around 100 µm and pressures that can reach several gigapascals (GPa), which, combined with film thickness less than 1 µm, lead to highly non-Newtonian behaviour. The situation is further complicated by thermal feedback whereby lubricant rheology dissipates heat causing an increase in temperature, which in turn affects the rheological properties of the fluid. Despite the importance of being able to reliably predict and optimise such behaviour, there is a lack of experimental data to support the modelling efforts in this area and, to the best of the authors’ knowledge, there is no effective method to accurately measure the distribution of temperature three-dimensionally within the oil film. This information would be useful in validating the disputed rheological models being used to predict friction [13]. The aim of this research is therefore to develop an efficient and reliable method to map the temperature distribution on both bounding surfaces and three-dimensionally through the thickness of the lubricant film of an EHD contact. To do this, a ball-on-disc apparatus and infrared camera are used to generate and detect temperature maps using an approach based on Winer’s and, more recently, Reddyhoff and co-workers’ studies [14].

Infrared thermography or so-called infrared thermal imaging is a spectroscopic technique based on the acquisition of electromagnetic infrared radiation. Experimental approaches typically involve an infrared camera with a microscope lens sensitive to IR radiation used to collect radiation emitted from samples, which is then processed to convert the radiation to temperature according to Planck’s law. The advantages of this infrared approach are accurate spatially resolved measurements, with high thermal resolution, and the ability to obtain transient variations and separate radiation components from the different components that make up the interface.

In 1950s, Bowden and Thomas [15] used a lead sulphide cell sensitive to transient infrared radiations to monitor the temperature of rubber surfaces; this is the first time an infrared technique was used in the field of tribology. After Bowden and Thomas, infrared techniques have been used as a thermal measurement in the field of tribology in a number of ways; for instance, in the 1970s, Winer and co-workers [16, 17] used it to measure temperatures in EHD contacts. Following this, a number of researchers have focused on the temperature distribution in EHD contacts by using similar techniques based on infrared microscopy. Examples are reported by Hou and Wen [18], Yagi and Nakahara [9, 19, 20], Spikes and co-workers [21–23], and Reddyhoff and co-workers [14, 24].

In 1995, Spikes and co-workers [22] used the infrared technique to record temperature maps of EHD contact surfaces and used these to calculate shear stress distributions according to Jaeger’s moving heat source theory [25, 26]. Following this, Spikes [27] further improved the technique by applying thin film coatings of chromium and aluminium onto transparent disc specimens in order to separate the radiations from both bounding surfaces.

Reddyhoff and co-workers [14, 24, 28, 29] overcame some of the limitations which Spikes encountered by employing a high spatial resolution (6 µm) and high sensitivity IR microscope lens to improve accuracy and map the contact temperature rises down to 0.01 K. They also validated the method by comparing the shear stress calculated from the temperature with the measured friction. Reddyhoff et al. [24] then extended the technique to measure asperity contacts by improving the calibration procedure and using a super-resolution method to further improve spatial resolution down to 1–2 µm [28, 30].

Despite these experimental studies and a number of thermal models built on different methods, such as multi-grid multi-level [31], to show the temperature variations, very few attempts have been made to obtain the temperature distribution within the lubricant film itself (as opposed to the temperatures of the bounding surfaces) under EHD conditions. This issue is tackled in the present contribution, where the IR technique has been developed to measure for the first time the temperature distributions of *both* contact surfaces and oil film in an EHD contact configuration. The detailed information of the proposed methodology is presented in Sect. 2 (Methodology), which highlights the features used to enhance existing IR methods to perform temperature mapping with unprecedented three-dimensional spatial resolution. Experimental results obtained using the proposed methodology are then presented and discussed in Sect. 3, where the newly developed technique is deployed to analyse typical EHD contact conditions and confirm the capability and validity of the method. Concluding remarks are presented in Sect. 4.

## Methodology

It has so far not been possible to use the IR technique to obtain oil film temperature distributions in EHD contacts accurately since variations in emissivity, which links the measured radiation to the temperature, have not been accounted for. The emissivity of a material is defined as the ratio of emissive power of a nonblack body at the surface to the emissive power of a black body at the same temperature [32, 33], which equals its absorptivity. However, emission from a fluid is a volumetric process which is a combination of reflection, transmission and absorption. In this case, the emissive power at the surface implies a small contribution of radiation from beneath the surface. Therefore, when thin films are considered (with thickness below a critical value), this subsurface contribution becomes important and the variation in emissivity with thickness must be considered. Based on the research reported in Ref. [34], the critical thickness of liquid, above which there is no variation in emissivity, is a few centimetres; this was also verified by simulation in Ref. [33], and means that in a thin EHD oil film, it is necessary to define a thickness–temperature-dependant emissivity. Therefore, the aim of this research is to extend the experimental IR technique by accurately characterising the emissivity of oil film as a function of both temperature and film thickness in order to obtain the temperature distribution within the oil film.

An interface was produced by loading a rotating steel ball half immersed in lubricant against a rotating sapphire disc, both mounted in a conventional EHD rig manufactured by PCS Instruments. The in-contact surfaces temperatures of ball and disc were obtained using the method described in Sect. 2.1 at locations within the contact region. The load for each test was controlled at 20 N giving a Hertz contact radius of 101 µm and a maximum and mean Hertz contact pressure of 0.926 and 0.583 GPa, respectively. The motion of the ball and disc were controlled by separate motors to obtain independent rotational speeds and slide–roll ratios (defined as ${\text{SRR}} = \frac{{U_{\text{disc}} - U_{\text{ball}} }}{{\frac{{\left( {U_{\text{disc}} + U_{\text{ball}} } \right)}}{2}}}$, where *U*_disc_ and *U*_ball_ are the surface speed of the disc and ball, respectively). The AISI 52,100 steel ball was 19.05 mm in diameter with roughness lower than 10 nm in each test. The transparent disc (made from single crystal Al_2_O_3_) has a diameter of 100 mm and a thickness of 3.5 mm. The roughness of the sapphire disc was lower than 10 nm, and similar to that of the steel ball. Santotrac 50 was used as the lubricant, which has a low viscosity and high traction coefficient under normal conditions (the dynamic viscosity of Santotrac 50 at 40 °C is 24.973 mPa s). This lubricant filled the reservoir to half-immerse the ball specimen in all tests, so that it was entrained into the contact by the rotation of the ball. The oil inlet temperature was monitored and controlled by a thermocouple probe located in the reservoir. This is acceptable since, according to [35], inlet shear heating in pure rolling conditions is negligible for entrainment speeds lower than ~ 3 m/s. As the emissivity of oil varies with film thickness as well as temperature, it was also necessary to measure film thickness over the whole contact region (using the Spacer Layer Imaging Method, SLIM on EHD rig, manufactured by PCS instruments) rather than just the average central film thickness.

**Fig. 1:**
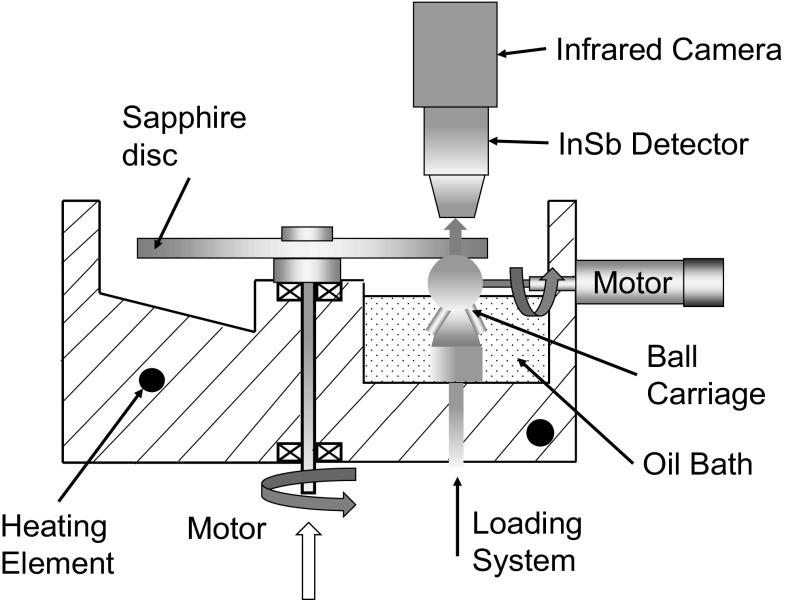
Schematic diagram of experimental apparatus

**Fig. 2:**
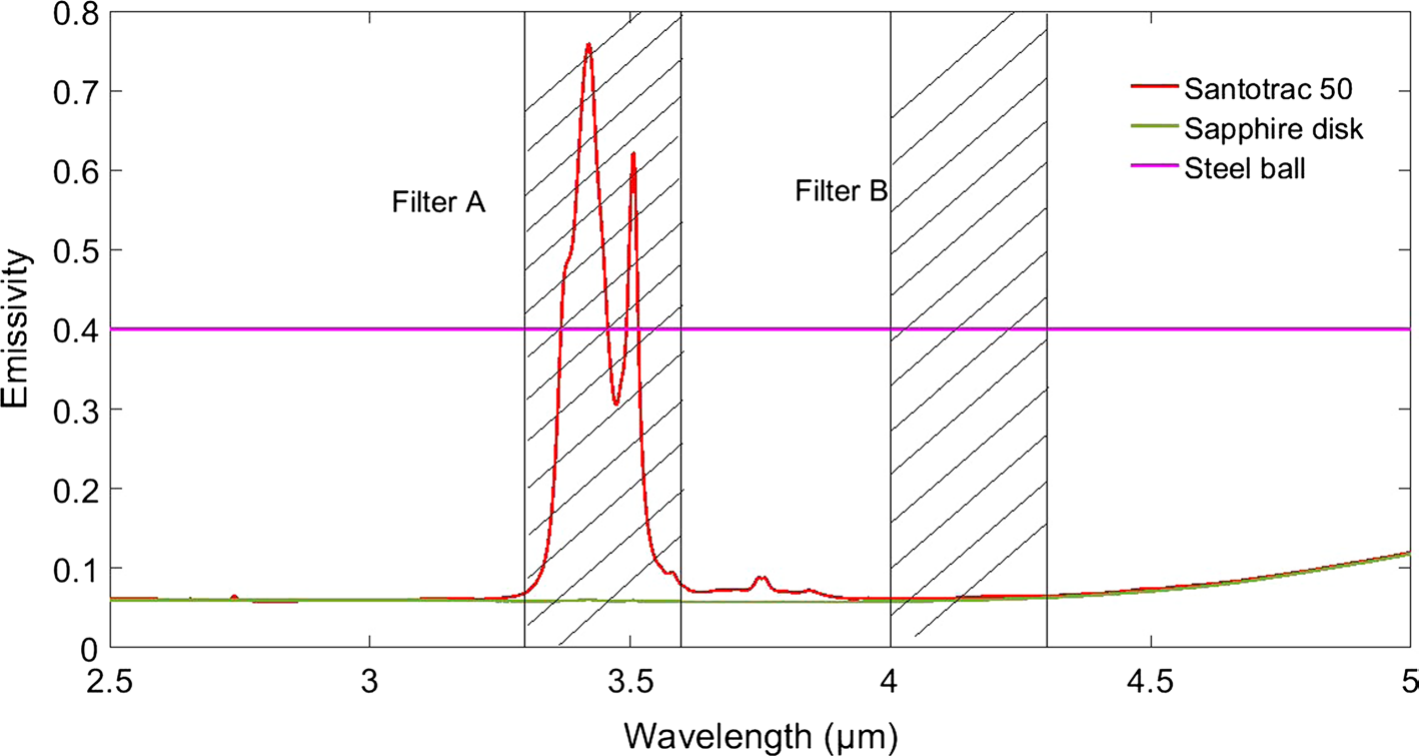
Spectra of emissivity/absorptivity of filters, sapphire disc, steel ball and Santotrac 50. Characteristic wavelengths of filters are shown as lines perpendicular to the *x*-axis. The transmission wavelength of filter A is from 3.3 to 3.6 μm, and filter B is from 4.0 to 4.3 μm as shown in the shaded regions of the graph

**Fig. 3:**
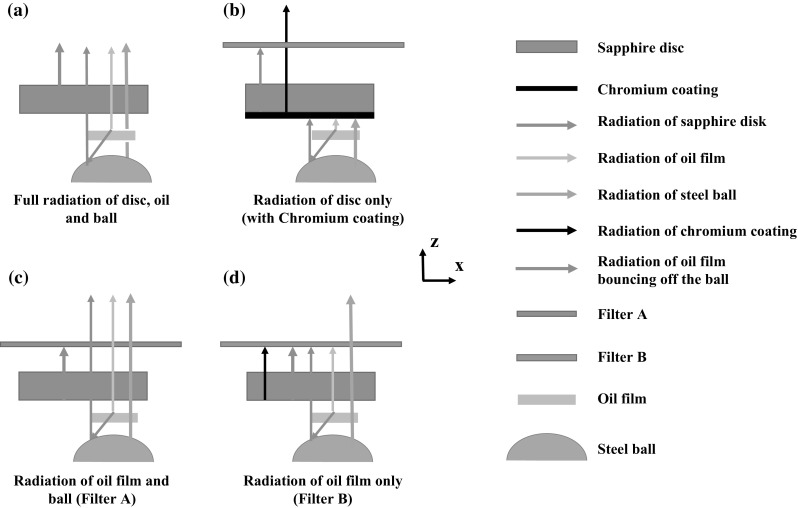
Schematic representation of the radiation components of the system: in **a** the radiation received by infrared camera is emitted by disc, oil and ball; in **b** the radiation from the oil and ball surface is reflected by the chromium coating (150 nm) so that only the radiation of the disc (chromium surface) can be received by infrared camera; in **c** the radiation of steel ball and oil can pass through filter A; in **d** only the radiation from steel ball can pass through filter B

An infrared camera (x6540s, FLIR, USA) was mounted above the rig and focused through the sapphire disc onto the contact interface to detect the infrared radiation emitted, as shown in Fig. 1. In order to reduce noise, 200 video frames were acquired and averaged for each test condition. The camera comprised an InSb detector, sensitive to radiation with wavelength range from 3 to 5 µm. According to absorptivity tests performed using an FTIR (Fourier transform infrared spectroscopy), the spectral range of Santotrac 50 emission is 3.3–3.6 µm, while the steel ball was shown to be a grey body which is defined as having a constant emissivity over all wavelengths at a given temperature, as shown in Fig. 2. According to Ref. [36], the sapphire disc only emits radiation at wavelengths above 4.3 µm. This means that the InSb detector is sensitive to radiation from the lubricant, the steel ball and the sapphire disc (see Fig. 3a) and it is necessary to measure the temperature of each. To achieve this, a method the involving two band pass optical filters and a chromium coating applied to the sapphire disc is employed as shown schematically in Fig. 3 and described below

1. A band pass filter (Filter B) with wavelength range 4.0–4.3 µm was used. With this filter in place, as shown in Figs. 2 and 3d, the radiation reaching the infrared camera originates only from the steel ball.
2. A second band pass filter (Filter A) with wavelength range 3.3–3.6 µm was used. As this filter lets through the wavelength range within which Santotrac 50 emits (see Fig. 2), the radiation received by the camera in this case consists of radiation emitted by both the oil film and the steel ball as illustrated in Fig. 3c.
3. A disc with a chromium coating, of thickness of 150 nm, applied to its lower surface was also used as shown in Fig. 3b. The effect of the chromium coating on the measurements was considered minor as, according to the research of Reddyhoff [29] and Jaeger [26], the temperature reduction across the 150 nm thickness coating was only about 10%. As chromium is opaque to infrared, the radiation from oil and ball is fully reflected so that the radiation received by the infrared camera in this case originates only from the lower surface of sapphire disc.

### Method to Obtain Temperature Distributions of Contacts

As described above, the separate radiation components from the system can be distinguished by using a combination of filters and a disc coating. The next step is to produce a calibration curve, which links the recorded emission to the temperatures of the components. However, this is challenging since relating emission and temperature requires knowledge of the emissivity, which for the oil is a function of both the temperature and layer thickness (since the thickness of the layers being measured is lower than the critical value of liquid as described in Ref. [34]).

To characterise the dependence of oil emissivity on temperature and film thickness, which is required to obtain temperature distributions from radiation data, a calibration procedure is applied which involves setting the ball and disc rotating against each other in pure rolling conditions and recording radiation with the method described above, while gradually increasing the oil temperature in the EHD reservoir from 25 to 80 °C. Since the heat generated in the contact under pure rolling conditions is typically less than 0.1 °C (due to a relatively small amount of shear and compression heating, which can be ignored [28]), this is equivalent to directly controlling the temperature of the oil film in the contact. This process is carried out twice; using an uncoated and a chromium coated disc. For each, recordings were made at different entrainment speeds and hence oil film thicknesses from 50 to 120 nm. This enabled the emissivity to be characterised as a function of both temperature and film thickness, as described below.

**Fig. 4:**
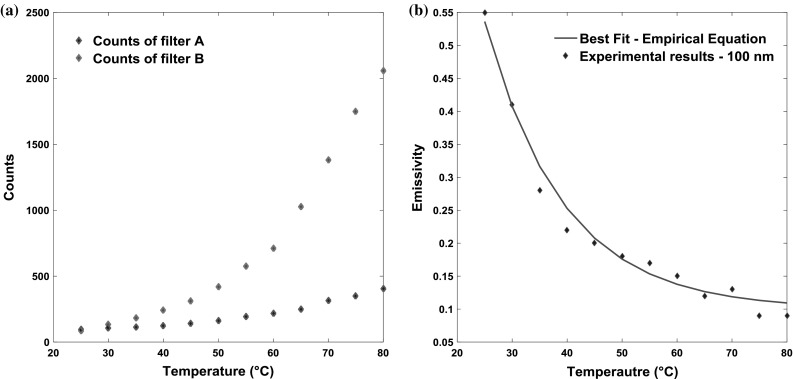
Counts and emissivity variations as a function of temperature at film thickness of 100 nm in pure rolling conditions

Figure 4a shows an example of the counts obtained by the infrared camera through filters A and B as the temperature of the oil is increased from 25 to 80 °C at a film thickness of 100 nm (1D arrays of emission data were also obtained at other film thicknesses).

According to Planck’s law and taking into account the radiation components shown in Fig. 3, the theoretical relationships between temperature and counts measured by the camera for filters A and B are given in Eqs. 1a and 1b, respectively. As shown in Fig. 3c, the radiation received by the infrared camera when filter A is used comprises three parts, namely the radiation from steel ball passing through oil film and filter A, the radiation from oil film passing through filter A and the radiation from oil reflected by steel ball surface and passing through oil film and filter A.

1a $$\begin{aligned} {\text{Counts}}_{\text{filter A}} & = \varepsilon_{\text{steel}} \times \rho_{{{\text{oil}}\left( {T_{\text{oil}} } \right)}} \times K_{{\left( {\text{filter A}} \right)}} \times \int_{3.3}^{3.6} {f_{\text{Steel}} \left( {\lambda ,T_{\text{steel}} } \right){\text{d}}\lambda } + \left( {\varepsilon_{\text{oil}} \times \tau_{\text{steel}} \times \left( {1 - \varepsilon_{\text{oil}} } \right)} \right) \\ & \quad \times K_{{\left( {\text{filter A}} \right)}} \times \int_{3.3}^{3.6} {f_{\text{oil}} \left( {\lambda ,T_{\text{oil}} } \right){\text{d}}\lambda } + \varepsilon_{\text{oil}} \times K_{{\left( {\text{filter A}} \right)}} \times \int_{3.3}^{3.6} {f_{\text{oil}} \left( {\lambda ,T_{\text{oil}} } \right){\text{d}}\lambda } \\ \end{aligned}$$

1b $${\text{Counts}}_{\text{filter B}} = \varepsilon_{\text{steel}} \times K_{\text{filter B}} \times \int_{4.0}^{4.3} {f_{\text{Steel}} \left( {\lambda ,T_{\text{steel}} } \right){\text{d}}\lambda }$$

where Counts_filter A_ and Counts_filter B_ are the counts received by camera passing through filters A and B, *K*_filter A_ and *K*_filter B_ are constants that depend on the properties of filters A and B, *ε*_steel_ is the emissivity of steel ball, *ρ*_oil_ is the transmissivity of oil, which is the measurement of the ability of oil to transmit infrared radiation, *ε*_oil_ is the emissivity of oil, *λ* is the wavelength and (*λ*, *T*) is a function of wavelength and temperature according to Planck’s law. Equations 1a and 1b have been simplified by eliminating both the emissivity of the steel surface and the transmissivity of the oil using the following theory of energy conservation, which in general can be written as:

2 $$\varepsilon + \rho + \tau = 1$$

where *ε* is the emissivity, *ρ* is the transmissivity, and *τ* is the reflectivity of material, which is the measurement of the ability of a material surface to reflect radiation. For a thermally opaque solid surface such as the steel ball, the transmissivity can be ignored so that *ε*_steel_ + *τ*_steel_ = 1, while for most liquids the reflectivity can be ignored so that *ε*_oil_ + *ρ*_oil_ = 1. Therefore, by inputting counts and temperatures obtained from calibration tests and rearranging, it is possible to obtain emissivity at different temperatures and film thicknesses as all the variables reported in Eq. 1 can already be determined given the characteristics of the filters and the steel ball (see values shown in Table 1) apart from the oil emissivity. The integral terms based on Planck’s law are the monochromatic blackbody emissive power which only depends on the wavelength of filters and temperature of the materials under investigation. Once a relationship between emissivity and temperature is obtained (for a given film thickness) as exemplified in Fig. 4b, it can be applied to emission measurements obtained during subsequent sliding tests to obtain the oil film temperature.

**Table 1:** Values of the constants used Eq. 1

Variables	Values
*ε*_steel_	0.4
*τ*_steel_	0.6
*K*_(filter A)_	1.0 × 10^−7^
*K*_(filter B)_	1.5 × 10^−7^

By changing the temperature and entrainment speed and repeating the calibration procedure using Eqs. 1 and 2, emissivity as a function of both temperature and film thickness can be obtained, to which the following Eq. 3 can be fitted:

3 $$\varepsilon_{\text{oil}} = h \times \left( {a + b \times e^{{ - c \times T_{\text{oil}} }} } \right)$$

where *h* is the film thickness of oil, *a*, *b* and *c* are the constant values obtained from curve fitting. The functional form Eq. 3 has been chosen in accordance with Planck’s law, for which the emissivity usually presents a rapid exponential decay and reaches an almost constant value for higher temperatures. This has been proved by Schopp et al. [37]. Once constants *a*, *b* and *c* have been determined, Eq. 3 can be substituted into Eq. 1, so that the only unknown values are the temperature of the components.

Tests were then run at different slide–roll ratios from 0.02 to 2 with a constant central film thickness of 100 nm, to characterise fully the temperature variations within the oil film under rolling/sliding conditions. In these tests, both experimental EHD apparatus and the test lubricant are the same as used for the calibration tests. The oil temperature in the EHD reservoir, and hence inlet temperature in the contact region, was maintained at 40 °C throughout the sliding tests. The emission measurements, recorded during these sliding tests, were then input into Eq. 1 so that the only unknown values are temperature of the ball and oil film (average through its thickness), can be found using an iterative process. Note: the disc surface temperature is obtained using a simple calibration procedure, since the chromium coating prevents radiation components from the disc and ball reaching the camera.

Due to the side and outlet constriction effects, the film thicknesses across the contact vary. To account for this, the calibration tests (defining the relationship between temperature vs. counts and emissivity) were obtained at a range of film thicknesses from 50 to 120 nm and the relevant calibration data were used based on the measured film thickness at each location (linear interpolation was used to obtain emissivity at film thickness values in between those used in the calibration).

### Method to Obtain the Temperature Profile Through the Thickness of the Oil Film

This section describes how the variation in temperature through the thickness of the film can be estimated based on the experimentally acquired ball surface temperature, disc surface temperature and the through-thickness average temperature of the oil. The method involves assuming the temperature variation can be represented by a polynomial function and then finding the constants that define the polynomial by applying a number of physical constraints.

Three main assumptions made in this analysis are:

1. Assumption: the temperature varies with film thickness in the form of a polynomial of order *k* as presented in Eq. 4:
  4 $${\text{Temperature}} = m_{k} \times z^{k} + m_{k - 1} \times z^{k - 1} + \cdots + m_{1} \times z + m_{0}$$
  where *z* is the distance through the thickness of the film such that *z* = 0 is the ball surface and *m*_0,1,…*k*_ are constants to be determined. This assumption of a polynomial temperature distribution has been made by a number of other researchers. For instance, Kazama et al. [38] conducted a two-dimensional energy equation to simulate the through-thickness temperature variation and predicted a quadratic profile. Moreover, in research by Sadeghi and co-workers [39, 40], the temperature profile as a function of viscosity, conductivity and sliding speed was obtained numerically under elastohydrodynamic conditions and was close to parabolic in shape. The temperature profile is constrained by continuity during the construction process, which decreases the uncertainty. Therefore, if a discontinuity point is present in the actual temperature profile where the temperature is close to maximum or boundaries provide a sharp transition in temperature, these will appear smoothed. Note: this method is currently being refined in order to cope with sharp transitions in temperature, and this will be documented in a subsequent publication.
  In our case, the oil can be thought of as comprising multiple layers dividing the film through its thickness, each having its own temperature value as shown in Fig. 5. Unlike the usual *x*–*y* in-plane view of the contact area, this *x*–*z* through-thickness view of the film cannot be accessed experimentally, since it is ~ 100 nm and therefore smaller than the deformation of the contact and the resolution of the infrared camera (6.3 μm).

**Fig. 5:**
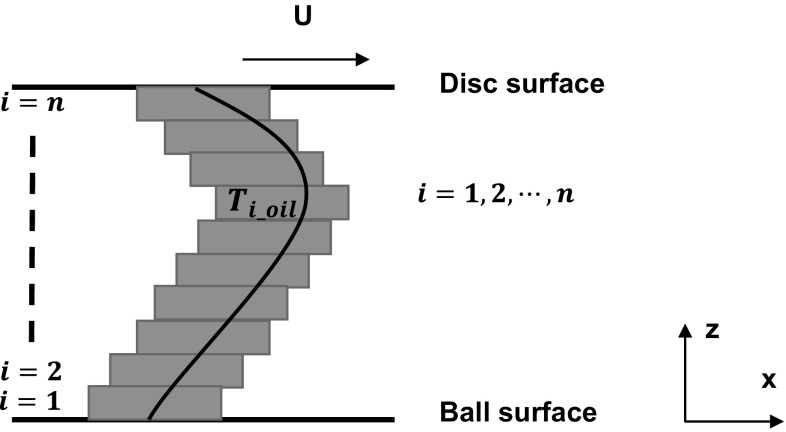
Schematic of the framework used to find through-thickness temperature profile
  Given an estimated temperature profile *T*_(*z*)_ as defined by Eq. 4, the theoretical infrared intensity of each layer can be described as in Eqs. 5 and 6.
  5 $$\begin{aligned} C_{i} & = \varepsilon_{\text{oil}} \left( {\frac{h}{n},T_{{i\_{\text{oil}}}} } \right) \times R_{\text{bb}} \left( {T_{{i\_{\text{oil}}}} } \right) \times \left( {1 - \varepsilon_{\text{oil}} \left( {\left( {n - i} \right) \times \frac{h}{n},T_{{n - i, {\text{oil}}}} } \right)} \right) \\&\quad + \varepsilon_{\text{oil}} \left( {\frac{h}{n},T_{{i\_{\text{oil}}}} } \right) \times \left( {1 - \varepsilon_{\text{oil}} \left( {i - 1} \right)} \right) \times \left( {1 - \varepsilon_{\text{oil}} \left( {h,T_{\text{oil}} } \right)} \right) \times R_{\text{bb}} \left( {T_{\text{oil}} } \right) \\ \end{aligned}$$
  6 $$C_{\text{total}} = \mathop \sum \limits_{i = 1}^{n} C_{i}$$
  where *i* is the number referring to a specific layer of fluid, *n* is the total number of layers, *T*_*i*_oil_ is the oil temperature at specific layer, *T*_oil_ is the average temperature of oil film, *h* is the film thickness of oil, *R*_bb_ is the radiation emitted by black body at the specific temperature, *C*_*i*_ is the photon counts of one layer of oil film, and *C*_total_ is the photon counts from the whole oil film. The integral of these layers is described as a discrete sum in Eq. 6 and corresponds to the total photon counts from the oil film received by the camera.
2. Assumption: all heat is generated by shear heating of the elastohydrodynamic film. According to the research by Reddyhoff et al. [28], there is only a small variation of temperature caused by compression heating and cooling compared with that caused by shear heating. This assumption is depicted in Eq. 7,
  7 $$\mathop {q_{\text{ball}} }\limits^{ \cdot } + \mathop {q_{\text{disc}} }\limits^{ \cdot } = q_{\text{total}} = \Delta \tau \times \bar{U}$$
  where ∆*τ* is the mean shear stress across the film thickness and $\bar{U}$ is the sliding speed. The shear stress will vary over the contact region, as a function of the pressure and the traction coefficient, but its average value can be estimated from,
  8 $$\Delta \tau = \mu \times \bar{P}$$
  where $\bar{P}$ is the average contact pressure and *μ* is the traction coefficient, which was independently measured on an MTM2, manufactured by PCS instruments, for slide roll ratios from pure rolling to pure sliding at a range of temperatures.
3. Assumption: All heat generated by shear is rapidly transferred to the bounding surfaces of the ball and disc by conduction only (i.e. there is negligible convection). This assumption is validated by the ratio of conduction to convection, shown in Eq. 9, according to Cameron [41]:
  9 $$\frac{{H_{\text{conduction}} }}{{H_{\text{convection}} }} = \frac{{k_{\text{oil}} }}{{\epsilon_{\text{oil}} \times \sigma_{\text{oil}} }} \times \frac{4D}{{\bar{U} \times h^{2} }}$$
  where the *k*_oil_ is the conductivity of the oil, $\epsilon_{\text{oil}}$ is the density of oil, *σ*_oil_ is the specific heat of oil, $\bar{U}$ is the entrainment speed, *h* is the film thickness, and *D* is the diameter of contact area in the convection direction. For Santotrac 50 at 40 °C with entrainment speed been set at 0.308 m/s, contact radius of 101 µm and film thickness of 100 nm, the ratio of conduction to convection is 65,000 and therefore convection can be safely ignored. If the local heat generation, *q*_total_ is calculated from Eqs. 7 and 8, the maximum temperature across film thickness *T*_max_ can be calculated directly, using conduction theory, combining Eqs. 10 and 11:
  10 $$- \,k_{\text{oil}} \times \frac{{T_{ \hbox{max} } - T_{\text{ball}} }}{{Z_{{T_{ \hbox{max} } }} }} = \mathop {q_{\text{ball}} }\limits^{ \cdot }$$
  11 $$- \,k_{\text{oil}} \times \frac{{T_{ \hbox{max} } - T_{\text{disc}} }}{{h - Z_{{T_{ \hbox{max} } }} }} = \mathop {q_{\text{disc}} }\limits^{ \cdot } ,$$
  where $Z_{{T_{ \hbox{max} } }}$ is the film thickness of oil at the position of maximum temperature, *T*_ball_ and *T*_disc_ are the temperature distribution of ball and disc which are obtained from the experiments described in Sect. 2.1.
  The thermal conductivity of the oil *k*_oil_ required in Eqs. 10 and 11 is the main source of uncertainty in analysing the temperature profile. The values used are those obtained by Larsson and Andersson in Ref. [42], who measured the conductivity of lubricants over the large range of pressures under elastohydrodynamic conditions. More specifically, since the thermal conductivity is sensitive to contact pressure, Eq. 12 is used in which the pressure is assumed to be that predicted by Hertz in order to reduce complexity.
  12 $$k_{\text{oil}} = k_{0} \times \left( {1 + \frac{{C_{1} \times \bar{P}}}{{1 + C_{2} \times \bar{P}}}} \right)$$
  where $\bar{P}$ is the mean pressure, the constant *k*_0_ is the thermal conductivity of Santotrac 50 at normal pressure which is assumed to be 0.104 W/mK, constants *C*_1_ and *C*_2_ are 1.85 and 0.5, respectively.
  13 $$z = 0\quad T_{\text{ball}} = d$$
  14 $$z = h \quad T_{\text{disc}} = a \times h^{3} + b \times h^{2} + c \times h + d$$

The experimental technique described above acquires the through-thickness average temperature of the oil film and the temperature distributions of two contact surfaces as the oil is sheared at different slide–roll ratios. This set of measured temperatures, along with the energy equation and Planck’s law, provides four physical constraints (corresponding to Eqs. 6, 7, 13 and 14) which can be applied to find the values of the constants that make up the assumed polynomial. The highest order of polynomial function that can be determined from this number of constraining equations is three (i.e. the temperature variation can at best be approximated by a cubic function). Therefore, Eqs. 13 and 14 have been specifically determined for a third-order polynomial. The process of obtaining temperature profiles using a third-order polynomial is presented as a flowchart in “Appendix,” Fig. 14. It must be noted that the temperature profiles obtained from the proposed methodology are bound to be more accurate than those obtained adopting quadratic polynomials and other methods which mostly assume the temperature profile to be a third-order polynomial but with maximum temperature at fixed position across the film thickness. The value of *n* defines the number of points and the accuracy of temperature profile. In this case, *n* was kept constant in all analysing process. A MATLAB script was built to input all the constraints and calculate temperature profile. This script was tested by generating a random temperature distribution, (i.e. picking random constants for Eq. 4) applying the methodology described in Fig. 14 to simulate measured values and then inputting these into the script to obtain the temperature profile which was the same as the one initially generated.

## Results and Discussion

**Fig. 6:**
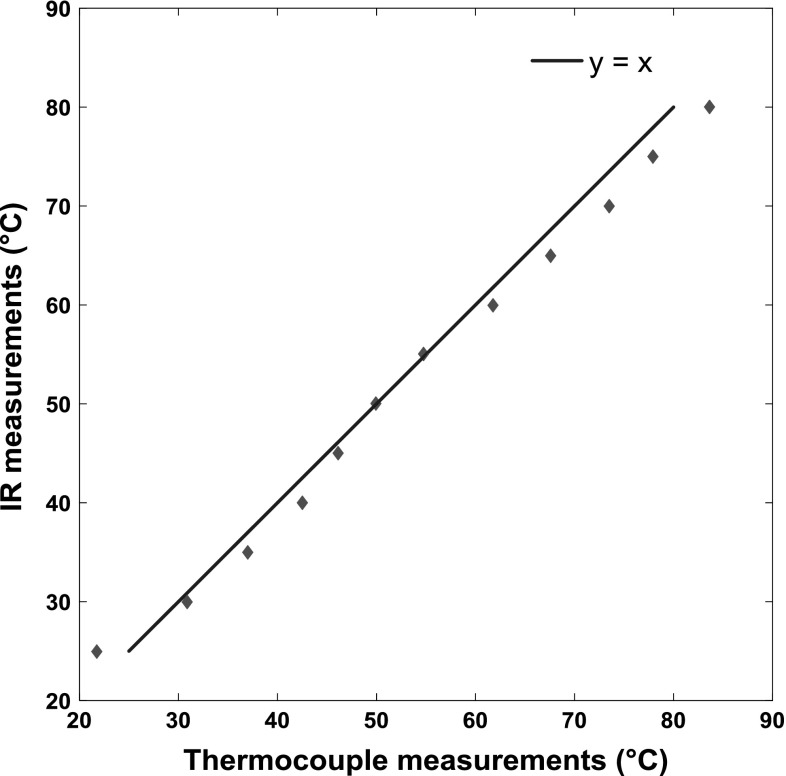
Comparison of average temperatures of the oil entrained at the inlet (measured using a thermocouple) and obtained from thermal camera measurements and Planck’s law

Figure 6 shows the comparison of the mean temperature of the oil entrained at the inlet (measured using a thermocouple) and the measurements obtained using the thermal camera and Planck’s law as described above. This test, in which the temperature was gradually increased, was conducted under pure rolling conditions, where the temperature in the contact equals that in the lubricant reservoir. For all measurements, the entrainment speed was adjusted so that the central film thickness was maintained at 100 nm. Reassuringly, temperature values calculated by using Planck’s law are close to those obtained directly using a thermocouple. This proves that the method presented in Sect. 2 can be effectively applied when the central film thickness is maintained at a stable value.

**Fig. 7:**
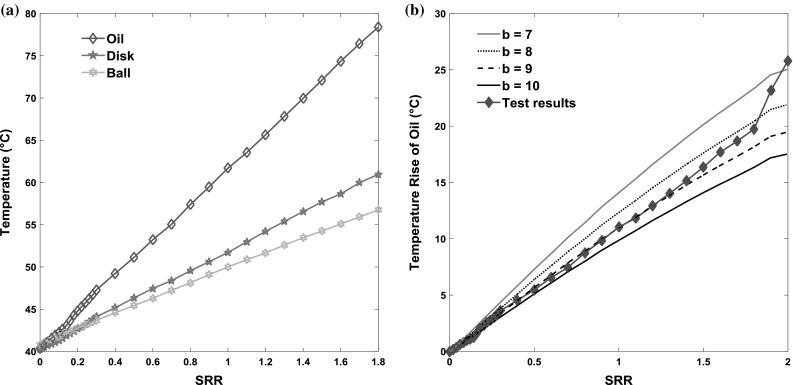
**a** Temperatures of oil and contact surfaces versus sliding–rolling ratio; **b** temperature rise of oil calculated from Eq. 15

Figure 7a indicates how the mean temperature of the oil film and contact surfaces vary with slide–roll ratio from 0 to 2 (with the disc surface moving faster than ball surface). It can be seen that the average temperature of the oil is significantly higher than either of the surfaces, which is to be expected due to the low conductivity of the film combined with the fact that within the film is where the heat is generated. It is also evident that there is a difference between the temperatures of the two surfaces. This is in agreement with studies by Manton and Cameron [43] and Reddyhoff et al. [14] and simulation study of Clarke et al. [44, 45], and is discussed below.

The data shown in Fig. 7a can also be used to test equations, based on theory originally developed by Carslaw and Jeagar [26] and Archard [46] that are commonly used in studies to calculate the oil film temperature, (e.g. see [27, 47, 48]):

15 $$T_{{{\text{rise}}\_{\text{oil}}}} = T_{\text{oil}} - T_{\text{s}} = \Delta T_{i} + \frac{{h\tau \bar{U}{\text{SRR}}}}{{bK_{\text{oil}} }}$$

where $T_{{{\text{rise}}\_{\text{oil}}}}$ is the temperature rise of oil film (note: in certain studies this refers to the maximum temperature through the thickness, and in others it refers to the average temperature), *T*_oil_ is the temperature of oil film, *T*_s_ is the average temperature of the two surfaces, ∆*T*_*i*_ is the temperature rise at inlet region, *h* is the film thickness of the oil film, *K*_oil_ is the conductivity of Santotrac 50 which is assumed to be 0.104 W/mK at 40 °C [27], *τ* is the mean shear stress of the oil film, and *b* is a constant which is suggested to vary broadly in the range between 4 and 24 [27], although other values have also been considered [48]. The differing choice of constant, *b*, should depend on two factors (1) whether *T*_rise*_*oil_ refers to the maximum or average film temperature, and (2) whether heat is assumed to be generated evenly throughout the thickness of the film (Couette flow) or just in the median plane.

The temperature difference between oil film and surfaces can be calculated using Eq. 15 inputting: the traction coefficient measured by MTM2, the measured temperature of the two surfaces and the measured film thickness. Note: as shown in Eq. 15, the average temperature rise in the film is proportional to the slide–roll ratio (SRR) and mean shear stress. Figure 7b shows the comparison between the temperature rise in oil film calculated in this way and that measured directly. As shown, there is good agreement between theory and experiment when the value of *b* in Eq. 15 is set to 9. According to Archard’s analysis [46] and considering we are measuring the average temperature within the film, a value of 12 would imply that the lubricant is subject to uniform shear through the film and a value of 8 would imply that shear is localised on the median plane. This therefore suggests that lubricant shear is neither due to pure Couette flow nor is it localised on the mid-plane. In this case, the heat condition theory cannot easily predict the temperature distribution within the film, since the distribution of shear/heat distribution is poorly characterised. It is, however, possible to infer the through-thickness temperature distribution from the experimental measurements, as will be demonstrated in the next section… It can also be seen from Fig. 7b, that there is a discrepancy between experimental results and theoretical results at high values of SRR. This may be because the oil reservoir does not stabilise at 40 °C under these high SRR conditions, since according to the temperature measurement by thermocouple during tests, when the value of SRR was set to 100%, the oil reservoir temperature was increased to 45 °C.

**Fig. 8:**
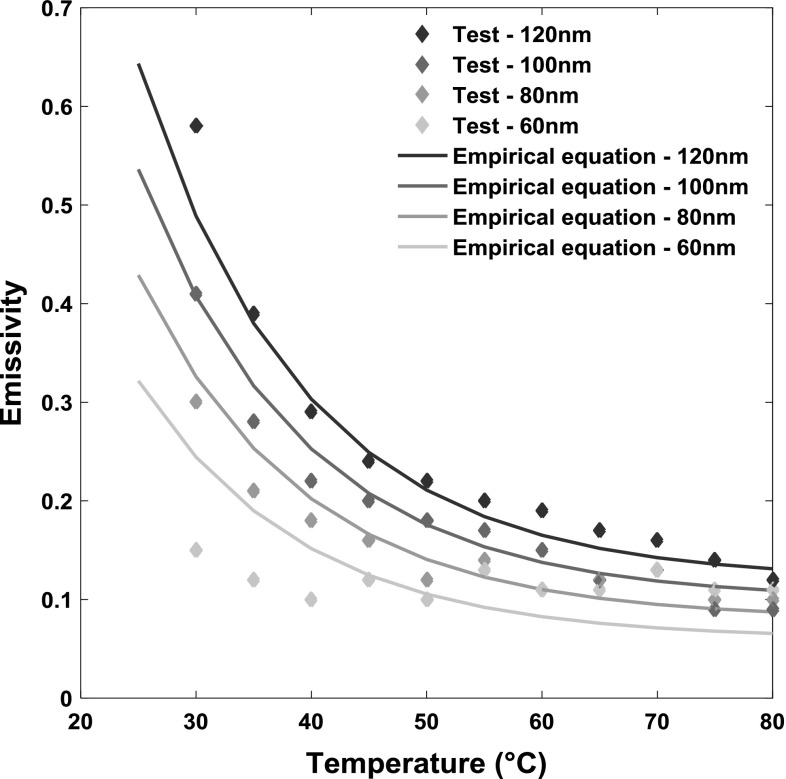
Emissivity of oil film as functions of temperature and film thickness (Color figure online)

The emissivity of oil film is obtained as a function of the temperature and the film thickness using the method described in Sect. 2.1. By fitting Eq. 3 to the emissivity values in Fig. 8, the values of the constants *a*, *b* and *c* were found to be 0.01, 5 × 10^6^ and 0.07, respectively. Note that the constants *a*, *b* and *c* were found by matching the curve in Fig. 4b and also reported in Fig. 8 for the 100 nm film. As shown in Fig. 8, the curve fitting lines computed using the empirical function in Eq. 3 agree well with experimental results also for film thicknesses of 80 and 120 nm. However, the result obtained for film thickness of 60 nm is not as well matched, and this is attributed to the uncertainty in the experimental measurements due to the low thermal radiation of oil film at low temperature and the high contribution from noise recorded by the infrared camera for very thin films.

The average film temperatures (averaged in *x*, *y*, *z* directions) presented in Fig. 10 were obtained at a constant film thickness of 100 nm. However, if the temperature distribution over the contact is required (showing variations in the *x*–*y* plane), it is necessary to apply the above technique to discretised points in the film, each with different thickness. This is why it was necessary in the calibration to define the emissivity, not just at different temperatures, but also at different film thicknesses. To this end, Fig. 8 presents the emissivities that were obtained from calibration tests when the temperatures of base oil ranged from 25 to 80 °C and film thicknesses from 60 to 120 nm. To the best of the authors’ knowledge, this is the first time the variation in emissivity of a liquid film with both temperature and film thickness has been simultaneously characterised. This verifies that, unlike solids, liquids are non-grey body with emissivities that are severely affected by local temperature. In Fig. 8, all emissivity curves with different film thickness show an initially rapid decrease with temperature and an approximately constant value for temperatures above ~ 70 °C. This trend is similar to the study of liquids presented by Schopp et al. [37] and also agrees with the results reported in [49], in which the emissivity or absorptivity of hydrocarbons or other liquids increased with film thickness for values up to 4.5 μm.

**Fig. 9:**
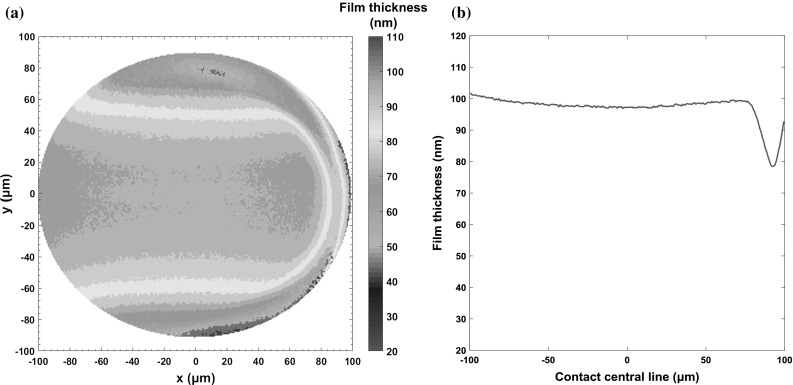
Film thickness distribution over contact area at speed of 0.308 m/s and the base oil of 40 °C, measured by SLIM on EHD 2

In order to select the correct emissivity at each point in the contact, it is necessary to measure the film thickness at each point in order to input in Eq. 3. As shown by the contour plot of central film thickness in Fig. 9a, this was achieved by SLIM tests under pure rolling conditions with an entrainment speed of 0.308 mm/s at an oil reservoir temperature of 40 °C. It can clearly be seen that the horseshoe region close to the outlet of the contact area contains lower film thickness values of approximately 80 nm compared to the central film thickness of 100 nm.

**Fig. 10:**
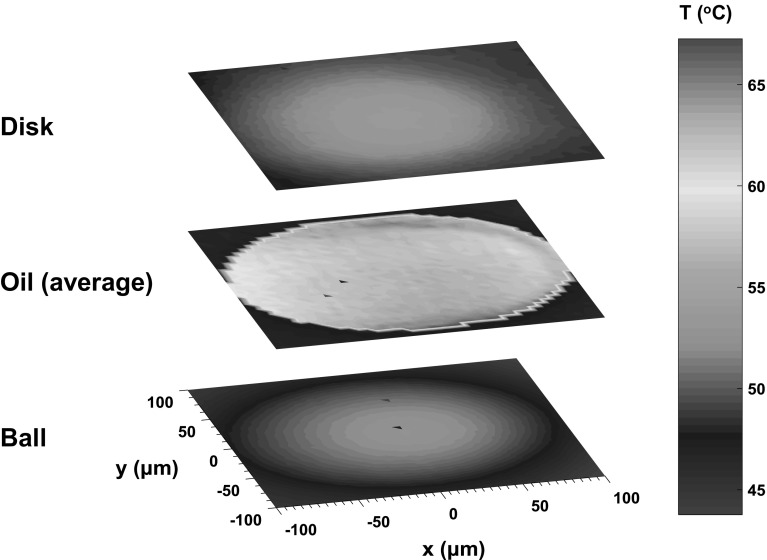
Temperature map of oil film and contact surfaces at SRR of 100% with inlet oil temperature of 40 °C

The in-contact surface temperatures of ball and disc were obtained using the method described in Sect. 2.1 at locations within the 101 µm radius contact region. A set of results obtained when the average film thickness was 100 nm with an entrainment speed of 0.308 m/s and a slide–roll ratio of 100% and temperature of 40 °C are shown in Fig. 10. Here, the temperature distribution of the oil film and the two bounding surfaces of the disc and ball have been obtained.

The maximum ball surface temperature is 52 °C. As the steel ball is mainly heated by the oil through conduction, the position of the maximum temperature on steel ball surface is slightly to the right with respect to the centre of the contact, i.e. closer to the outlet. It is interesting to note that the temperature of the steel ball remains low and close to the oil reservoir temperature outside the contact and around the side lobes, where the film thickness is minimum. Moreover, the maximum temperature shows good agreement with the measurements reported in [14] obtained under the same conditions. The temperature maps of ball and disc surfaces in Fig. 10 are similar to each other with values increasing to the centre of the contact and then decreasing. There is only a slight difference between the two temperature maps which is that the maximum temperature of disc surface is higher than ball surface due to the conductivity of sapphire disc being considerably lower than the steel ball.

As expected, the highest temperatures in oil film are generated at the lowest film thickness region as shown in Fig. 10. Specifically, temperatures over 60 °C are found at the side lobes and the exit region along contact centre line which correspond to the horseshoe region of film thickness distribution shown in Fig. 9. The oil reservoir temperature was 40 °C, however, there were no temperatures measured below 55 °C with the oil entering the contact from the inlet having already heated up to 55 °C due to the high shear rate experienced in the inlet. Although the maximum temperature of oil was 67 °C, the temperatures of oil increased from inlet to maximum no more than 12 °C during the fluid’s transit through contact region.

**Fig. 11:**
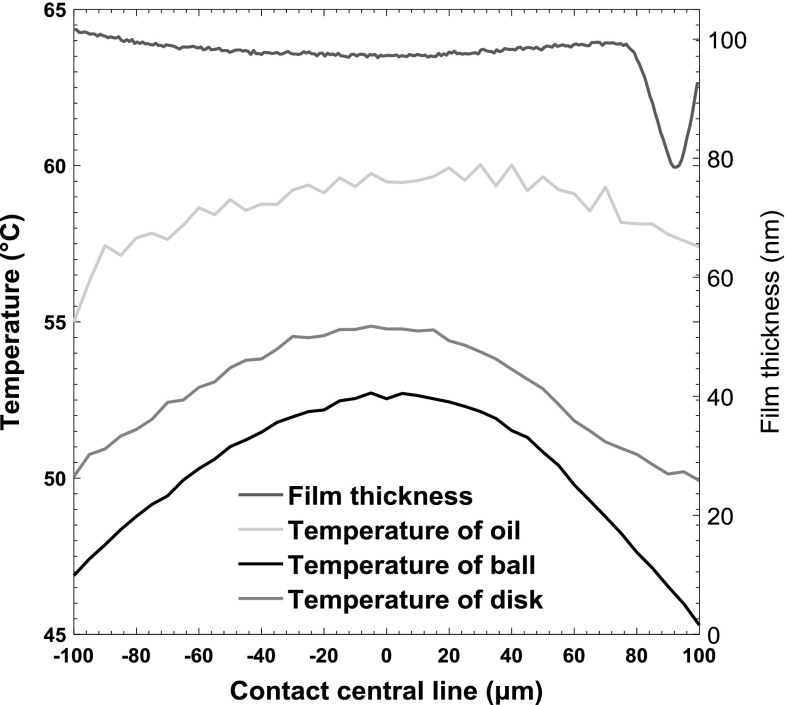
Film thickness and temperatures of oil (average) and surfaces along the central line of the contact

Figure 11 shows the ball, disc and average oil temperatures, as well as oil film thickness along the centre of the contact when the central film thickness is 100 nm, for an entrainment speed of 0.308 m/s and SRR of 100%. In this case, the fluid shear rate is approximately 3.08 × 10^6^/s. The relatively small temperature rise of oil across contact region also proves that most of the heat is conducted to the ball and disc through oil in the direction of film thickness and the heat convection has minor impact on the heat transfer. It can also be seen that both the ball and disc surface temperatures decrease rapidly after the point where the oil temperature is a maximum so that the temperature difference between oil and contact surfaces grows rapidly. Lower film thickness, on the other hand, increases the shear rate of fluid (according to in $\dot{\gamma } = \frac{\nu }{h}$, where *v* is the sliding speed and *h* is the film thickness) which increases the oil temperature.

**Fig. 12:**
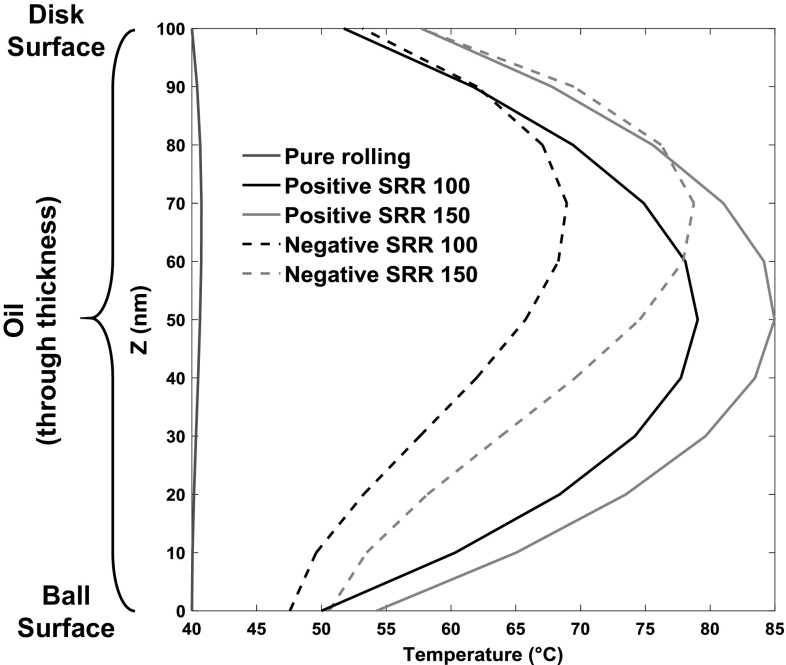
Temperature profiles through the film thickness obtained using a third-order polynomial at different slide–roll ratios. The dashed lines represent temperature profiles at negative slide–roll ratios with the sapphire disc surface moving faster than steel ball; the solid lines present the temperature profiles at positive slide–roll ratios with the steel ball surface moving faster than the sapphire disc

The temperature profiles through the film thickness in the *z* direction are shown in Fig. 12 for the cases of different slide–roll ratios of − 150%, − 100, 0, 100 and 150%. For a given speed, the positive slide–roll ratio indicates the sapphire disc surface is moving faster than steel ball and vice versa. It should be noted that the ball surface is always defined as the position within the film distance/height equals zero. It can be seen from the figure that the temperatures of the oil through the film are strongly affected by the slide–roll ratio. For a given constant entrainment speed and inlet oil temperature of 40 °C, as the sliding speed increases, the total energy generated in the oil increases according to Eq. 7, which causes an increase in lubricant temperatures. In all these cases except pure rolling, the temperature of the disc is higher than that of the ball surface since the thermal diffusivity $\alpha = \frac{k}{{\rho \times C_{p} }}$ and effusivity $\sqrt {k\rho C_{p} }$ of steel being much higher than sapphire. According to the research of Carslaw and Jaeger [25, 26], who simulated a semi-infinite body moving under a heat source, the main factor of heat transfer within the body when the surface is moving with high speed is conduction. Conversely, when the surface speed is low, the convection of heat dominates. Therefore, when the disc has high speed and low conductivity, the temperatures of sapphire surface are higher than that of the steel ball. In addition, when the ball surface moves faster than the disc with conduction dominant, more heat is removed by ball so that the location of the maximum temperature of lubricant within oil film approaches the sapphire disc surface, while the temperature of ball at negative SRRs is lower than at positive SRRs.

**Fig. 13:**
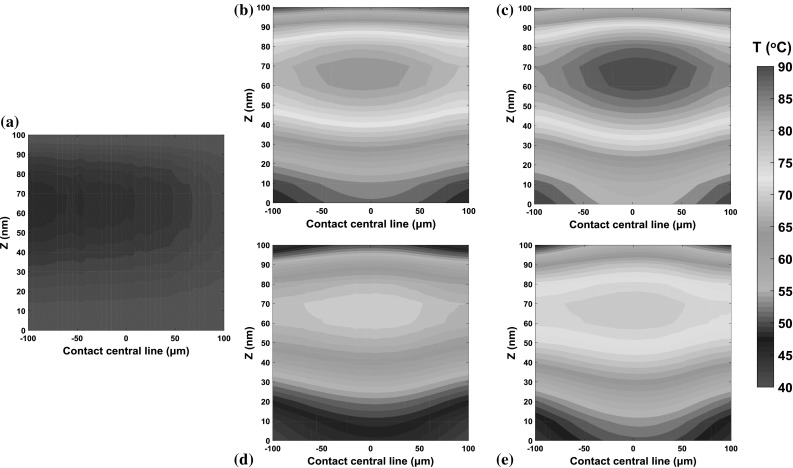
Temperature maps through the thickness of the oil film along the centre of the contact, for **a** pure rolling, **b** SRR = 100%, **c** SRR = 150%, **d** SRR = − 100%, **e** SRR = − 150%

**Fig. 14:**
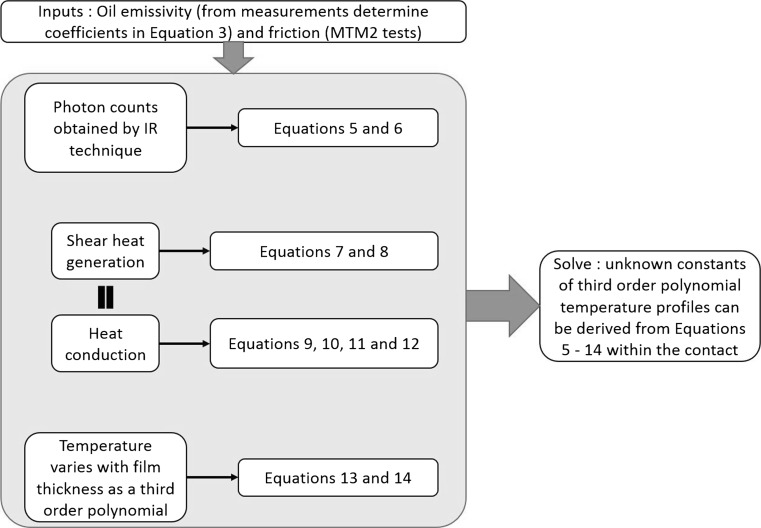
Flowchart summarising the calibration procedure and methodology adopted for 3D temperature mapping in EHD lubrication

The temperature distributions of oil within the film are shown in Fig. 13 under the same conditions as those in Fig. 12, but here the variations in the *x* direction are shown instead of the average values along the entraining direction. It can be seen in Fig. 13a that the maximum temperature occurs at the inlet region in pure rolling due to the heat of compression as there was no shear heating under these circumstances. This shows good agreements with the simulation work presented by Kim and Sadeghi [39] and Habchi and Vergne [50, 51]. Furthermore, the temperature distribution under pure rolling conditions remains close to the oil reservoir temperature of 40 °C due to the negligibly small contribution of compression heating in agreement with Reddyhoff et al. [28]. In contrast, the maximum temperatures of the oil at high slide–roll ratios occur close to the contact centre and significant temperature rises are observed, compared with those obtained under pure rolling conditions. The maximum temperature of the oil film is 50 °C higher than the oil reservoir for a SRR of 150%, while that of the ball and disc surface are only 15 °C higher. The peak and average temperatures of oil at negative SRRs are lower than at positive SRRs because, ignoring heat convection in the *z* direction, more heat is conducted away by the faster moving, conductive ball.

## Conclusion

An experimental method to measure the thermal behaviour of lubricant and surfaces within an elastohydrodynamic circular/point contact under rolling/sliding conditions has been successfully developed. This uses an infrared microscope and band pass filters, and analyses data based on Planck’s law and the energy equation. The result is that variations in temperature can be resolved in the *x*, *y* and *z* directions, which provides data to help understand the rheological behaviour of lubricants under high pressure. The main conclusions can be summarised as follows:

1. The emissivity of the oil film, when its thickness is in the nanometre range, is strongly dependant on temperature and film thickness. Specifically, the emissivity reduces with decreasing film thickness and increasing temperature. This must be characterised in order to measure oil temperature correctly.
2. The temperatures of the surfaces and the oil film increase with slide–roll ratio due to increasing shear rate. However, within the contact, the temperature rise within the oil increases at the side lobes of horseshoe region where the oil film thickness is lower than its average value, and hence the shear rate is higher. Under sliding conditions, the maximum temperature of the oil in the *x* direction (averaged through the thickness) occurs close to contact centre.
3. The disputed constant value present in commonly used equations to predict mean oil film temperature rise has been investigated and the value of 9 obtained. Considering the average temperature was measured, this suggests that the lubricant in question is not subject to purely Couette flow and that shear is localised on a non-median plane. Thus, a more involved theoretical treatment is necessary to predict oil film temperatures subject to localised shear, which is the focus of the next publication.
4. In all cases measured, the through-thickness temperature profile is largely determined by heat conduction in the *z* direction. This results in the temperature of the insulating sapphire disc being always higher than that of the conductive steel ball. Furthermore, the maximum temperature of the oil within the film is located closer to the sapphire disc surface for negative slide–roll conditions (i.e. then the ball is moving faster than disc). This is because the conductive ball is able to remove more heat in this case.

This measurement technique will be used in future to study thermal behaviour of different lubricants and to validate and inform CFD (e.g. [52, 53].) and other numerical EHL (e.g. [39, 50]) simulations.

## Appendix

See Fig. 14.

## List of symbols

$K_{{{\text{filter}}\,3.3{-}3.6}}$: Integral properties of transmissivity of sapphire, surface area of one pixel, quantum efficiency, transmissivity of filter A, reflectivity of filter A and integration time
$K_{{{\text{filter}}\,4.0{-}4.3}}$: Integral properties of transmissivity of sapphire, surface area of one pixel, quantum efficiency, transmissivity of filter B, reflectivity of filter B and integration time
${\text{Counts}}_{{{\text{filter}}\,3.3{-}3.6}}$: Counts received by camera passing through filter A
${\text{Counts}}_{{{\text{filter}}\,4.0{-}4.3}}$: Counts received by camera passing through filter B
*D*: Diameter of contact area
Filter A: Band pass filter with wavelength from 3.3 to 3.6 μm
Filter B: Band pass filter with wavelength from 4.0 to 4.3 µm
*f*(*λ*, *T*): A function of wavelength and temperature in Planck’s law
*h*: Oil average central film thickness
K_oil_: Conductivity of test lubricant
$\bar{P}$: Average contact pressure
$\mathop {q_{\text{ball}} }\limits^{ \cdot }$: Heat generation of ball surface
$\mathop {q_{\text{disc}} }\limits^{ \cdot }$: Heat generation of disc surface
*q*_total_: Heat generation of oil
$\bar{U}$: Entrainment speed of lubricant
*T*_ball_: Temperature of ball surface
*T*_disc_: Temperature of disc surface
$Z_{{T_{ \hbox{max} } }}$: Film thickness of oil at the position of maximum temperature
*ε*: Emissivity
*τ*: Reflectivity
*ρ*: Transmissivity
*λ*: Wavelength of filters
*μ*: Traction coefficient of oil
Δ*τ*: Average shear stress of oil film
$\epsilon_{\text{oil}}$: Density of oil
*σ*_oil_: Specific heat of oil
